# Delivery of Antisense Oligonucleotides Using the Nano‐Cell Vesicle Technology System (nCVTs) for Targeted Cancer Therapy

**DOI:** 10.1002/smll.202509094

**Published:** 2025-12-16

**Authors:** Yi Hsuan Ou, Wei Heng Chng, Ram Pravin Kumar Muthuramalingam, Prathyusha Raghunathan, Nichakan Khunkitchai, Choon Keong Lee, Jae Ha Jun, Zi Xiu Ng, Phua Tian Xin, Wei Jiang Goh, Jiong‐Wei Wang, Bertrand Czarny

**Affiliations:** ^1^ Department of Pharmacy and Pharmaceutical Sciences Faculty of Science National University of Singapore 18 Science Drive 4 Singapore 117543 Singapore; ^2^ Department of Surgery Yong Loo Lin School of Medicine National University of Singapore 1E, Kent Ridge Road, NUHS Tower Block Singapore 119228 Singapore; ^3^ School of Materials Science and Engineering College of Engineering 50 Nanyang Avenue, Block N4.1 Singapore 639798 Singapore

**Keywords:** biomimetic hybrid system, cell derived drug delivery system, gene delivery, gene therapy, nucleic acid therapeutics

## Abstract

Nucleic acid therapeutics (NATs) promise to revolutionize the fight against diseases like cancer, yet their full potential remains untapped due to significant delivery challenges. In this study, innovative nano‐cell vesicle technology systems (nCVTs) are developed to target NATs to cancer cells. nCVTs are created by fusing cationic lipids with pre‐emptied cell membranes that can efficiently bind and transport NATs such as antisense oligonucleotides (ASOs). In the study, nCVTs loaded with an anti‐cancer ASO, AS014 (nCVTs_AS014_) outperformed both liposomes and free AS014 in delivering the therapeutic payload into cancer cells, reaching both the cytoplasm and nuclei with remarkable efficiency while displaying lower intrinsic cytotoxicity. Most impressively, nCVTsAS014 demonstrated potent inhibition of tumor growth in vivo, underscoring its potential as a powerful cancer therapy platform. Altogether, the findings highlight nCVTs as a promising strategy to unlock the therapeutic power of NATs, paving the way toward more effective and targeted treatments for cancer.

## Graphical abstract

Nano‐Cell Vesicle Technology (nCVTs) integrates cationic lipids with tumor‐homing cell membranes to achieve efficient intracellular transport of antisense oligonucleotides. The system enhances nuclear delivery, minimizes cytotoxicity, and suppresses tumor growth in vivo, establishing nCVTs as a versatile and biocompatible platform for next‐generation nucleic acid cancer therapeutics.

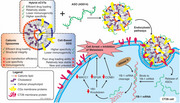

## 1 Introduction

Along with the ever‐increasing understanding of the molecular mechanisms of cancer pathogenesis and therapeutic resistance, the use of nucleic acid therapeutics (NATs) for the treatment of cancer has gained popularity in the past decades.^[^ ^1^, ^2^, ^3^ ^]^ NATs aim to correct the genetic defects by modulating the gene expression levels of target cells.^[^ ^4^ ^]^ Through sequence‐specific binding, most NATs can manipulate the target gene expression in cells with high specificity.

Despite the potential of NATs in cancer treatment, their therapeutic efficacy is limited by their low specificity, poor uptake and rapid clearance from the blood circulation.^[^ ^5^ ^]^ Most NATs have poor physicochemical properties, which hinder effective delivery to intracellular target sites. As such, NATs often require additional chemical modifications and a drug delivery system (DDS) for effective therapeutic properties. Indeed, chemically modified oligonucleotides have shown to impart stability and improve pharmacokinetic profiles of oligonucleotides.^[^ ^6^ ^]^ Alternatively, DDSs deliver the NATs to their intended site of action while conferring protection against nucleases.^[^ ^7^ ^]^ Currently, the choice of DDS for most gene delivery applications has revolved around the use of viruses, lipid‐based nanoparticles (such as liposomes and lipid nanoparticles), polymer‐based nanoparticles and *N*‐acetylgalactosamine (GalNAc) conjugation. While viruses are known for their high transduction capability and natural tropism toward specific cells, safety concerns, including immunogenicity and carcinogenicity, remain elusive.^[^ ^8^, ^9^ ^]^ On the other hand, non‐viral delivery vectors, which are considered safer with a larger genetic payload and relative ease of synthesis, have lower transfection efficiency and lack specificity for targeted delivery.^[^ ^10^ ^]^ Therefore, the development of a specific and efficient gene delivery system is required for broader clinical applications of NATs.

Recent progress in lipid‐based delivery systems has underscored the importance of rational formulation optimization for achieving tissue‐specific accumulation of antisense oligonucleotides (ASOs).^[^ ^11^, ^12^ ^]^ Guan et al. introduced a tailored delivery platform for an ASO therapy against hepatocellular carcinoma, leveraging a rationally designed lipid system to enhance potency and precision.^[^ ^11^ ^]^ The authors engineered uniform nanoparticles by encapsulating CT102 ASO within a neutral cytidinyl lipid (DNCA) and a cystine‐based cationic lipid (CLD). This cytidinyl/cationic lipid pairing conferred stability, efficient cellular uptake, and selective in vivo accumulation in the liver. These nanoparticles achieved nuclear delivery within ≈2 h, accelerating target engagement and effectively downregulating IGF1R mRNA. In a subsequent work, Pan et al. systematically screened lipid compositions based on in vivo biodistribution, optimizing the lipid ratios of the cytidinyl/cationic lipid platform (known as DCP) with markedly improved liver‐targeting capacity.^[^ ^12^ ^]^ The DCP system comprising DNCA, CLD, and DSPE‐PEG with flexibility in the component ratios exploited multiple intermolecular interactions, including hydrogen bonding, π–π stacking, and electrostatic forces with oligonucleotides, achieved over 60% hepatic accumulation. Furthermore, the integration of chemical modifications, such as 2′‐O‐methoxyethyl (2′‐MOE) and glycosyl conjugation (such as GluNAc) to form Glu‐CT102_MOE5_, enhanced serum stability, cellular uptake, and antitumor efficacy against hepatocellular carcinoma. Collectively, these findings highlight how in vivo‐guided formulation design that balances encapsulation efficiency, surface physiochemistry, and oligonucleotide chemistry can substantially improve targeted ASO delivery.

Inspired by these investigations on ASO delivery, here we describe the development of a novel gene delivery platform termed cationic nano‐cell vesicle technology systems (cationic nCVTs). As a hybrid system, nCVTs are postulated to represent a synergistic combination of both synthetic and cell‐derived DDS: the synthetic lipid components impart structural integrity, ease of functionalization, and efficient complexation while the cell membrane components provide nCVTs with intrinsic targeting ability and improved cellular uptake by target cells without the need for additional PEGylation. nCVTs were previously developed by our group through the fusion of neutral lipids with pre‐emptied cells (i.e., cell ghosts (CGs)).^[^ ^13^, ^14^ ^]^ As an additional development, in this work nCVTs were produced using cationic lipids that could be complexed with the highly negatively charged NATs. Exploiting the intrinsic homing capability of U937 monocytic cells toward inflammatory and tumor sites, cell membrane components from U937 CGs were used to enhance the selectivity of cationic nCVTs toward cancer cells.^[^ ^15^, ^16^ ^]^ By preserving the cell membrane features of U937 monocytes, cationic nCVTs are expected to maintain these targeting properties and thus enhance the efficacy of gene therapy in comparison to cationic fusogenic liposomes (LIPO).

In this study, we loaded cationic nCVTs with AS014, an anti‐cancer antisense oligonucleotide (ASO). AS014 has been reported to effectively knockdown the expression of Y‐box binding protein 1 (YB‐1) in lung cancer cells.^[^ ^17^, ^18^ ^]^ YB‐1 is a transcriptional regulator involved in promoting cell survival through overcoming cell cycle arrest caused by DNA damage. As an oncogene, YB‐1 is upregulated in multiple cancers, thus often identified as a potential biomarker and therapeutic target.^[^ ^19^, ^20^, ^21^ ^]^ Downregulation of YB‐1 has been shown to inhibit tumorigenesis in many disease models such as colon cancer,^[^ ^9^, ^10^, ^11^, ^12^, ^13^, ^14^, ^15^, ^16^, ^17^, ^18^, ^19^, ^20^, ^21^, ^22^, ^23^ ^]^ neuroblastoma^[^ ^24^ ^]^ and breast cancer.^[^ ^25^ ^]^

It has been demonstrated that a high proportion of colorectal cancer (CRC) tissues have an elevated YB‐1 mRNA expression and that poor tumor differentiation, deeper tumor invasion, lymph node metastasis, and advanced Dukes' classification were all strongly correlated with higher YB‐1 protein expression in CRC.^[^ ^26^ ^]^ Hence, silencing YB‐1 expression in cancer cells is an attractive option to cause cell arrest and prevent malignant transformation. Through our study, we demonstrated how our cationic nCVTs could effectively deliver AS014 to target CT26 murine colon cancer cells and induced a strong anticancer effect.

## 2 Results and Discussion

**Figure 1:**
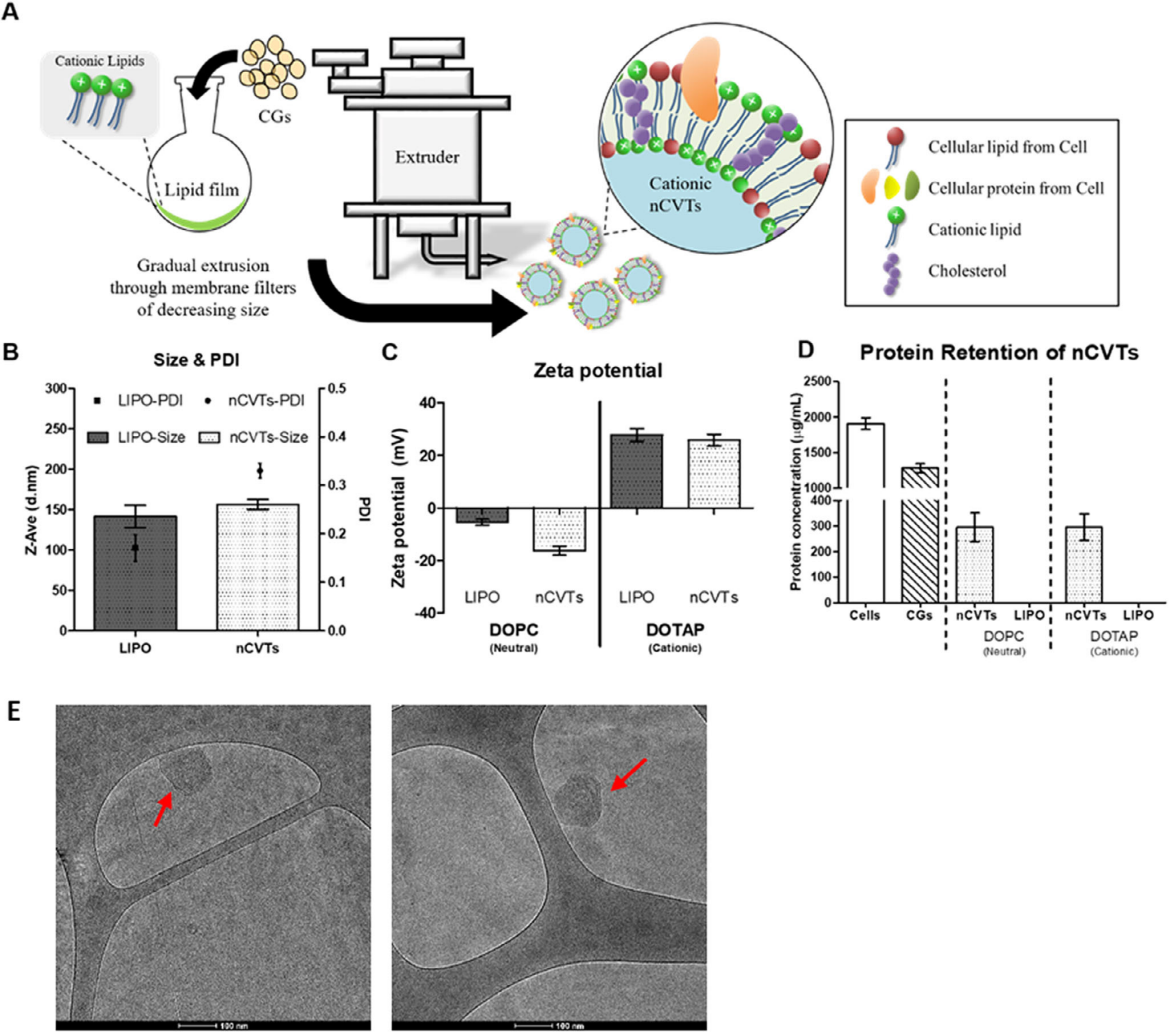
Schematic representation and characterizations of Cationic nCVTs. A) Experimental set‐up for cationic nCVTs production. 1 × 10^7^ CGs were used to rehydrate the cationic lipid film. The rehydration mixture was subsequently sonicated and extruded to produce cationic nCVTs. B) Hydrodynamic sizes and polydispersity indexes (PDI) of cationic nCVTs and LIPO. C) Zeta potentials of cationic nCVTs and LIPO formulations. The cationic formulations were compared to the neutral formulation produced using DOPC instead of DOTAP. D) Protein concentrations of respective nCVTs and LIPO formulation. The protein concentrations of cells, CGs and nCVTs were compared with cationic nCVTs, while liposomal formulations served as negative controls. E) Cryo‐TEM images of cationic nCVTs. Scale bar indicates 100 nm.

**Figure 2:**
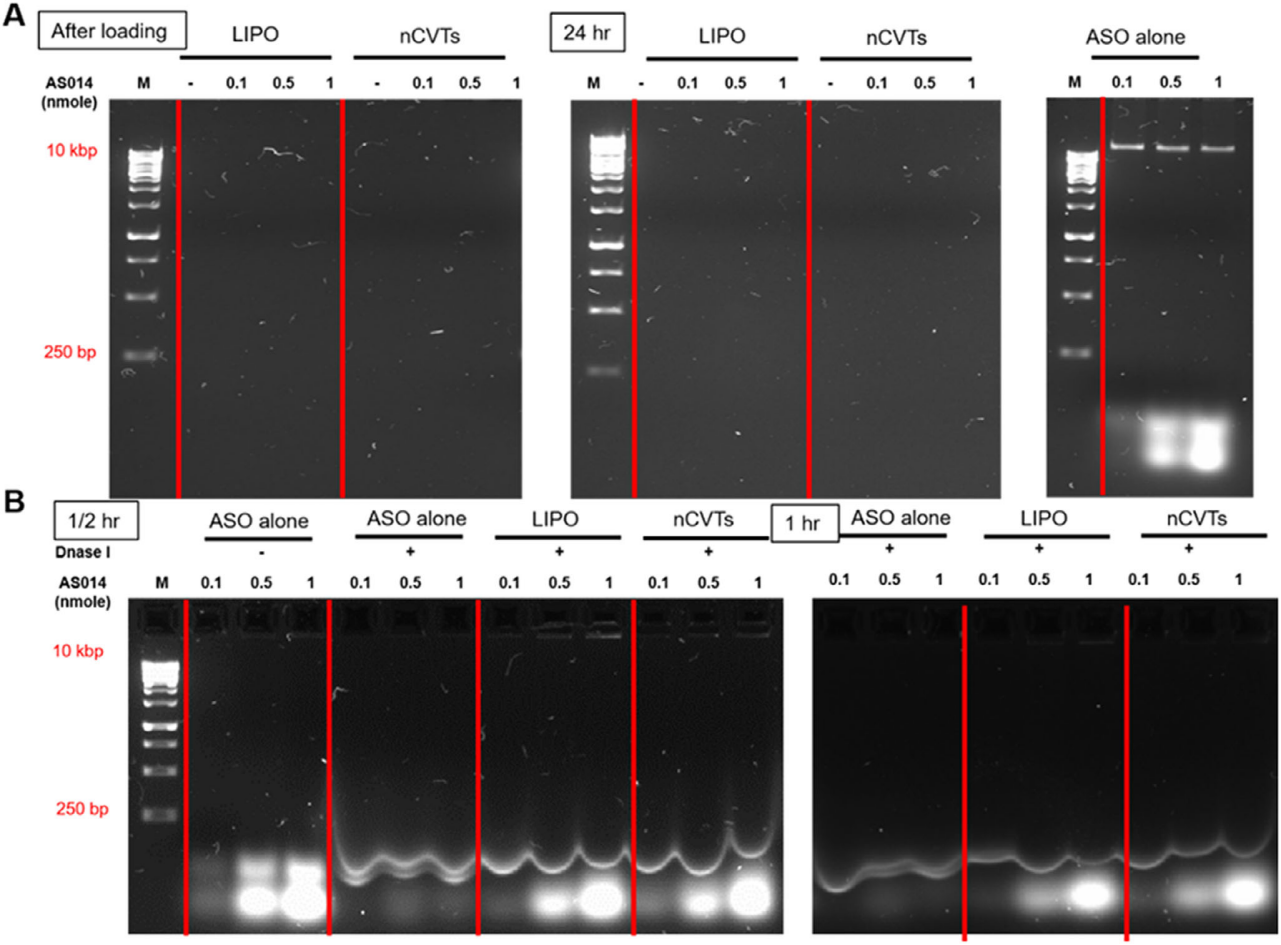
Agarose gel electrophoresis experiments on A) complexation and loading stability of nCVTs and LIPO, at different molar ratios, immediately after loading, and after 24 h of storage at 4 ^°^C. ASO alone was used as the control to indicate free (unbounded) ASO; B) Protection assay of ASO loaded onto nCVTs and LIPO against DNase I after incubation of 30 min and 1 h, respectively. Free ASO was used as a control to indicate the DNase digestion of free ASO. The DNA molecular ruler is indicated with m.

In order to prepare nCVTs, CGs were first produced from U937 cells using a previously established protocol.^[^ ^13^ ^]^ Monocytic U937 cells were chosen for production due to their intrinsic tumor targeting capability.^[^ ^15^ ^]^ The CGs suspension was used to rehydrate the cationic lipid thin film. The dispersion was then sonicated and extruded to facilitate the fusion of the cell membrane with the lipid components, and to reduce the size of the vesicles to the nano‐scaled range (**Figure** **1**A). CGs, instead of cells, were used for nCVTs production, because large aggregates and polydispersed vesicle populations were formed when cells were directly used in fusion with cationic lipids (Figure S1A, Supporting Information). Furthermore, when cells are used to form the hybrid, the resultant DOTAP‐cell hybrid (DOTAP_Cell_) had a negative zeta potential with similar protein retention as nCVTs (Figure S1B, Supporting Information). The negative zeta potential limits the ability of DOTAP_Cell_ to be complexed with the negatively charged NATs. This was demonstrated by the inability of DOTAP_Cell_ to deliver ASO in a cellular uptake study (Figure S2, Supporting Information). In comparison, CGs were significantly cleared of their intracellular contents (most of the cytoplasmic and nuclear contents), thus removing the potential confounders that could hinder the fusion process. In addition, the removal of the intracellular contents also improved the reproducibility and overall safety of the cationic nCVTs. The resultant cationic nCVTs were observed to have hydrodynamic sizes below 200 nm (Figure 1B), a desirable size range to potentially take advantage of the enhanced permeability and retention effect and avoid premature clearance by the reticuloendothelial system. As expected, the incorporation of DOTAP cationic lipids produced nCVTs with an overall positive zeta potential. These nCVTs had a positive charge (≈+30 mV), which was comparable to the cationic LIPO made from the same lipids and devoid of the cellular component (Figure 1C). Having a positive zeta potential allows nCVTs to easily load and complex with the negatively charged NATs. In this case, cationic nCVTs could be efficiently complexed with our ASO of interest, AS014. Cationic nCVTs were found to retain >25% of proteins from CGs, an amount comparable to our neutral nCVTs (Figure 1D). Cryo‐transmission electron microscopy (Cryo‐TEM) images also demonstrated that the cationic nCVTs were spherical vesicles of size ≈150 nm (Figure 1E, in correspondence of the red arrow). Moreover, proof of fusion assay (Figure S3A,B, Supporting Information) and fluorescence resonance energy transfer (FRET) assay (Figure S3C,D, Supporting Information) were conducted to confirm the successful fusion of the cell membrane components of CGs within the lipid bilayer. We also confirmed that key proteins markers expressed on the cell membrane of U937, such as CD63 and CD11a (which is part of lymphocyte function‐associated antigen 1 (LFA‐1, CD11a/CD18)) are retained on the nCVTs produced (Figure S3E, Supporting Information). Parallel proteomics studies on U937 immune cells, CGs, and nCVTs revealed that more than 227 functional proteins were consistently present across all three sample types (data not shown), confirming that key functional proteins in the whole cells (source of CGs) were preserved in the CG component as well as in the final formulation (nCVTs). This is in alignment with our previous finding that integrins targeting cell adhesion molecules (CAMs), typically found in monocytic cells, were retained in our nCVTs, supporting the intrinsic targeting capability of nCVTs toward diseased areas that overexpress CAMs.^[^ ^11^ ^]^ Moreover, including cationic lipids, such as DOTAP, enhanced surface charge and likely improved ASO loading efficiency due to stronger electrostatic interactions with the negatively charged oligonucleotides. Nonetheless, to determine the ability of nCVTs to efficiently complex with ASO, an electrophoretic mobility assay was performed. Increasing concentrations of ASO were added to LIPO and nCVTs. A complete retardation of ASO was observed for both LIPO and nCVTs formulations (**Figure** **2**A). There were no free ASO in the presence of cationic vesicles, as compared to the free ASO at different concentrations, which showed the characteristic fluorescent DNA bands with the binding of SYBR green dye. A similar trend was observed for complexes after 24 h of storage at 4 °C. This suggests that both LIPO and nCVTs were able to efficiently complex with ASO and the resultant ASO formulations were stable for at least for 24 h under storage at fridge conditions.

In addition to the ability to efficiently and stably complex with ASO, it is also crucial for a delivery system to protect its load against possible enzymatic degradation, particularly the DNase degradation. The formulations were tested against DNase I‐induced digestion at 37 °C, while free ASO was used as a control to monitor the integrity of the ASO. Expectedly, free ASO was digested by DNase I (Figure 2B). In contrast, LIPO and nCVTs protected the ASO from degradation by DNase I, as evidenced by the presence of fluorescent bands of ASO.

Of note, the ASO used in this study has phosphorothioate modification at both 5′‐ and 3′‐ ends, which are expected to increase its enzymatic stability. Despite the chemical modification, it was observed a partial digestion of AS014 after treatment with DNase I (i.e., faint fluorescent bands observed for ASO alone in Figure 2B). This emphasizes the need for a carrier (i.e., complexation of ASO with nCVTs and LIPO) to provide additional protection and preserve the integrity of ASO before reaching the target sites. Here, we demonstrated that nCVTs displayed a comparable complexation efficiency and capacity as LIPO, and at the same time were able to protect the loaded ASO against the degradation induced by DNase I (Figure S4, Supporting Information).

**Figure 3:**
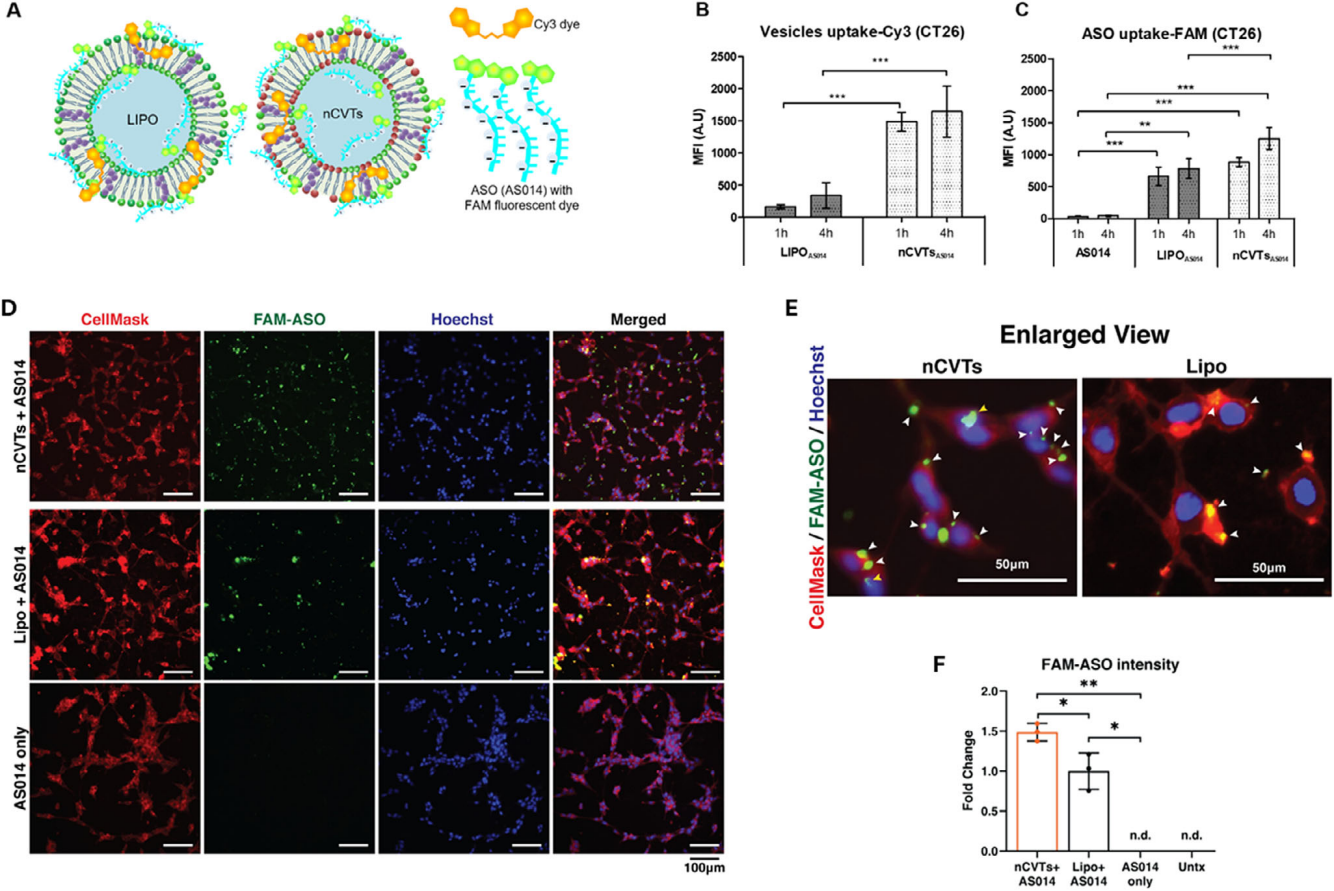
Cellular uptake of cationic formulations and ASO. A) Schematic illustrations on labelling and ASO loading of nCVTs and LIPO. B) Fluorescence assisted cell sorting (FACS) quantitative analysis of cellular uptake of LIPO (blue) and nCVTs (green); and C) uptake of ASO by CT26 cells treated with AS014 alone and LIPO_AS014_ and nCVTs_AS014_ at respective 1 and 4 h time points. ^**^ denotes *p* < 0.01 and ^***^ denotes *p* < 0.001 D) Confocal microscopy images of cells treated with respective formulations (nCVTs_AS014_, LIPO_AS014_ or AS014 only) for 6 h. Cell membranes were stained with CellMask DeepRed (red), cell nuclei were stained with Hoechst 33 342 (blue), and AS014 was tagged with FAM fluorescence (green). Scale bars indicate 100 µm. E) Enlarged confocal images of cells treated with LIPO_AS014_ and nCVTs_AS014_ for 6 h. Scale bars indicate 50 µm. F) Fold change of FAM fluorescence normalized to LIPO_ASO14_ fluorescence intensity. *N* = 3, n.d. = not detected, untx = untreated.

To investigate the in vitro cellular uptake of ASO, the vesicles were labelled with Cy3 monoester and the ASO was tagged with FAM fluorophore to facilitate detection (**Figure** **3**A). The formulations were then incubated with CT26 cells for 1 or 4 h prior to flow cytometry analysis. As shown in Figure 3B, the cellular uptake of nCVTs and LIPO was not time dependent. Instead, cellular uptake of both nCVTs and LIPO peaked at 1 h and subsequently plateaued (at the 4 h time point). The observed cellular uptake suggested that both nCVTs and LIPO exhibited a faster initial rate of cellular uptake, and nearing equilibrium, slowed, probably due to the depletion of receptors or degradation of the endocytosed vesicles over time. Although both cationic vesicles showed a similar trend in the cellular uptake kinetics, nCVTs displayed a significantly higher cellular internalization (in terms of Cy3 signal) than LIPO, which may be attributable to the preservation of membrane components from CGs, which mediated the cellular uptake of nCVTs (Figure 3B). To demonstrate the robustness of nCVTs in delivering the therapeutic cargo into the target CT26 cells, we examined the cellular uptake profiles of ASO for both nCVTs and LIPO samples. Similarly to the cellular uptake profiles of the vesicles, ASO levels were found to be significantly higher in CT26 cells incubated with nCVTs than LIPO over the same time periods. The improved cellular uptake of nCVTs was able to translate into an enhanced delivery of ASO to the cells, as higher cellular uptake of FAM‐ASO was observed in the cells treated with AS014‐loaded nCVTs than AS014‐loaded LIPO, and free AS014 is undetectable (Figure 3C). This increase in uptake of ASO can have a tremendous impact on its biological activity, as several reports in the literature demonstrated that an increase of cellular uptake by ≈10 folds can lead to over 1 000 folds of increment in the activity.^[^ ^27^, ^28^ ^]^

Further reinforcing the ability of a robust DDS to deliver its therapeutic cargo, confocal microscopy images also showed a clear uptake of FAM‐ASO when LIPO and nCVTs were incubated with CT26 cells for 6 h (Figure 3D). These data were consistent with the flow cytometry analysis, as the images showed a higher cellular uptake of ASO‐loaded nCVTs than ASO‐loaded LIPO in the FAM channel. Moreover, cells treated with nCVTs_AS014_ showed localization of FAM fluorescence in the nuclear region, suggesting that ASO can be delivered also to the nuclei (Figure 3E,F). In comparison, while LIPO could deliver ASO into the target cells, albeit at a lower concentration, nuclear delivery of ASO was not observed.

**Figure 4:**
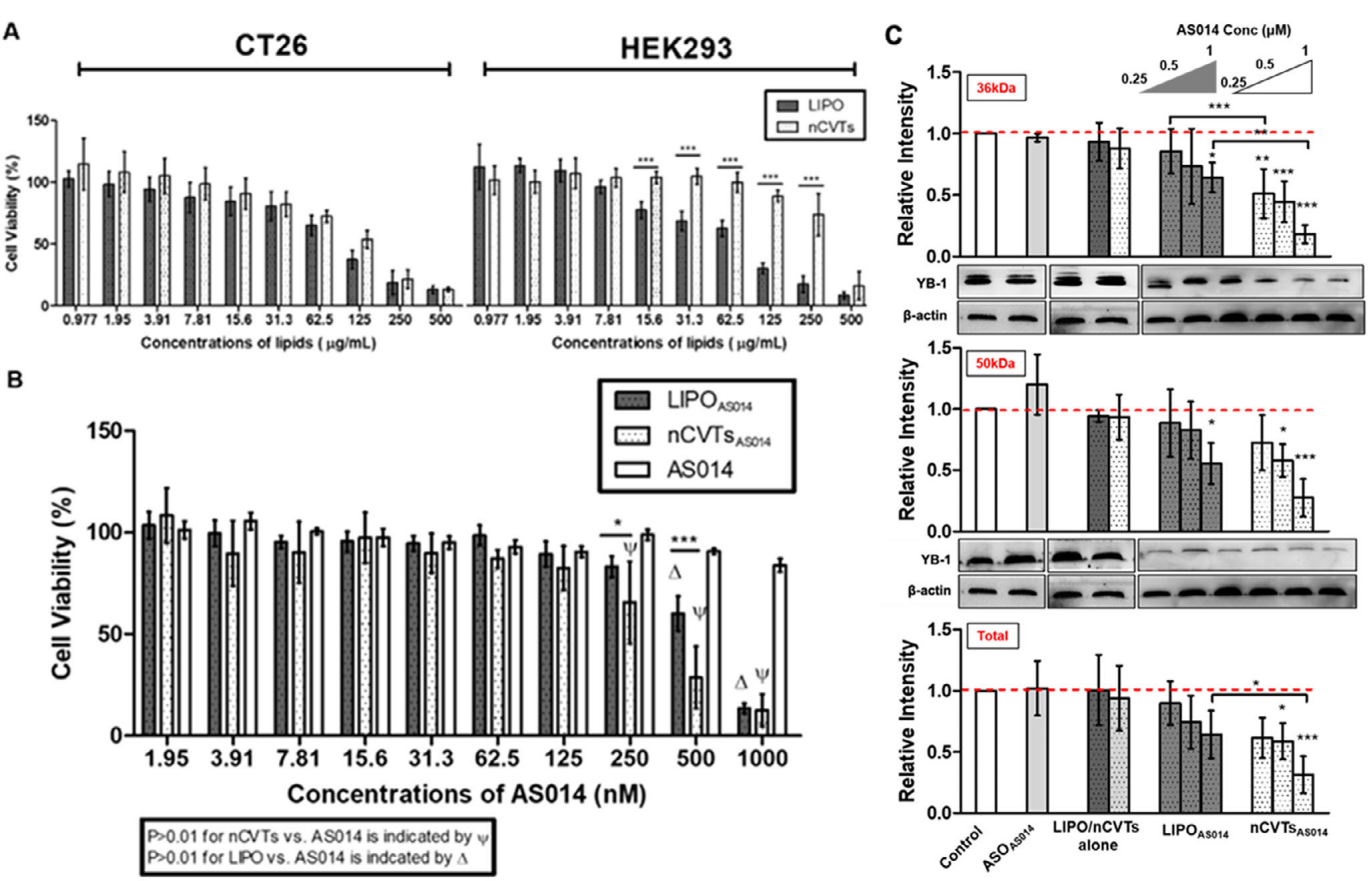
Comparison of nCVTs and LIPO on cytotoxicity and in vitro ASO delivery efficacy. A) Effect of empty cationic nCVTs and LIPO on the viability of CT26 and HEK‐293 cells after incubation for 72 h. B) Effect of AS014 and AS014‐loaded formulations on the viability of CT26 cells. C) Expression of YB‐1 protein in CT26 after 72 h incubation with LIPO_AS014_, or nCVTs_AS014_ analyzed by western blot. The relative intensity of the band was quantified using ImageJ and normalized to b‐actin (housekeeping protein) to ensure consistent comparison between the different blots. Cells without treatment, treated with free ASO and treated with empty LIPO or nCVTs were used as negative controls. β‐actin was used as a housekeeping protein. *N* = 3, data given as mean ± SD. ^*^ *p* < 0.05, ^**^ *p* < 0.01, ^***^ *p* < 0.001.

We also investigated the effects of the nCVTs *vis‐à‐vis* LIPO on the cell viability of CT26 (a cancer cell line) and HEK293 (a non‐cancer cell line). As expected, the high positive zeta potential of both LIPO and nCVTs contributed to some degree of cytotoxicity, particularly at the higher concentrations (i.e., 500 µg mL^−1^ of lipids), in both cell lines (**Figure** **4**A). When tested on CT26, both LIPO and nCVTs induced a similar degree of cytotoxicity throughout the various concentrations tested. In comparison, when tested on HEK293 cells, nCVTs displayed a vastly different profile from LIPO: only at the higher concentrations (i.e., 500 µg mL^−1^ of cationic lipids) nCVTs were cytotoxic toward HEK293 cells, but as the nCVTs concentration decreased, the cytotoxic effect declined drastically, becoming negligible at 125 µg mL^−1^ of cationic lipids. This lies in stark contrast to LIPO, which only became less cytotoxic below the concentration of 7.81 µg mL^−1^ of cationic lipids. Interestingly, when the difference in the cell viability between two cell lines was compared, we observed that nCVTs had a certain extent of preferential toxicity toward cancer cells. We attributed this phenomenon to the presence of cell membrane components from U937 (a monocyte cell line) on nCVTs, which enable nCVTs to utilize monocytes’ natural ability of targeting cancer cells. This testifies on the monocytes’ homing ability toward cancer cells, also proven in another cell‐based delivery system tested in a co‐culture of a cancer cell line and a non‐cancer cell line.^[^ ^16^ ^]^ This implies that nCVTs are expected to cause less collateral damage to the surrounding healthy cells, indicating a clear advantage of nCVTs over LIPO.

To investigate the effectiveness of our vesicles in delivering ASO to the target, both nCVTs_AS014_ and LIPO_AS014_ were incubated with CT26 for 72 h. The selected concentrations of the tested cationic vesicles had little or negligible effect on cell viability, to ensure that the biological activity of AS014 was not influenced by intrinsic effects on cell viability (Figure S5, Supporting Information). Without the use of a delivery system, AS014 alone showed little killing effect on CT26 due to its limited ability to permeate the cell membrane. Conversely, both AS014‐loaded formulations showed greater effect on the cell viability of CT26 (Figure 4B). nCVTs_AS014_ were shown to be more effective in reducing the cell viability of CT26 than LIPO_AS014_, especially at ASO concentrations of 250 and 500 nm. The improved activity of AS014 after loading onto nCVTs was postulated to be due to the higher cellular uptake of nCVTs than LIPO, as shown earlier, which facilitated and enhanced the delivery of AS014 into the cells, leading to a greater reduction in cell viability.

We also hypothesize that nCVTs undergo endosomal/lysosomal escape following endocytosis, allowing delivery of ASO while avoiding lysosomal degradation. Confocal imaging revealed that liposomes were largely colocalized with lysosomes from as early as 1 h post‐uptake and remained so up to 24 h, suggesting gradual degradation over time (Figure S6A, Supporting Information). In contrast, nCVTs showed minimal colocalization and were distributed in other intracellular regions at 1 and 4 h. Even after 24 h, a portion of nCVTs remained unassociated with lysosomes (Figure S6A, Supporting Information).

The quantification of Pearson's correlation coefficient reveals that liposomes showed a trend of higher colocalization with lysosomes as compared to nCVTs across all time points, suggesting a higher susceptibility to lysosomal degradation (Figure S6B, Supporting Information). This lower trend of colocalization of nCVTs in the lysosomes compared to liposomes suggests some extent of evasion from lysosomal degradation, even though future work will need to confirm this trend.

Finally, to elucidate the effective delivery of AS014 into CT26 by nCVTs, a western blot analysis of the YB‐1 protein was carried out (Figure 4C; Figure S7, Supporting Information). YB‐1 protein tends to migrate to 50 kDa in SDS‐PAGE,^[^ ^29^, ^30^ ^]^ while the proteolysis product of YB‐1 protein that is predominately found in the nucleus was reported as the band at 36 kDa.^[^ ^31^ ^]^ Free AS014‐treated cells showed no difference from untreated control, confirming that ASO alone has limited capability of entering cells and exerting its biological effect of knocking down YB‐1 protein expression. Both nCVTs_AS014_ and LIPO_AS014_ formulations were able to significantly lower the YB‐1 protein expression in CT26 cells as compared to the controls, especially at a higher concentration of AS014 (1 µm). Notably, the lower expression of the proteolysis product of YB‐1 protein (as indicated by the reduced signal observed at the 36 kDa mark) implies that nCVTs_AS014_ were able to significantly reduce the nuclear translocation of YB‐1 as compared to LIPO_AS014_. Translocation of YB‐1 from the cytoplasm to the nucleus happens when cells suffer from DNA damage, as nuclear YB‐1 is able to regulate the pro‐survival genes and activate genes responsible for drug resistance and cancer development.^[^ ^32^ ^]^ Since nuclear YB‐1 might be a predictive factor for the pro‐survival cellular response of cancer cells, the ability to downregulate the expression of YB‐1 protein in the nucleus highlights the potential of this gene delivery system. We demonstrated the successful and more effective knockdown of YB‐1 protein in CT26 cells with the treatment of nCVTs_AS014_. These results confirm the ability of our formulations to effectively deliver biologically active ASO into the target cells and knockdown YB‐1.

**Figure 5:**
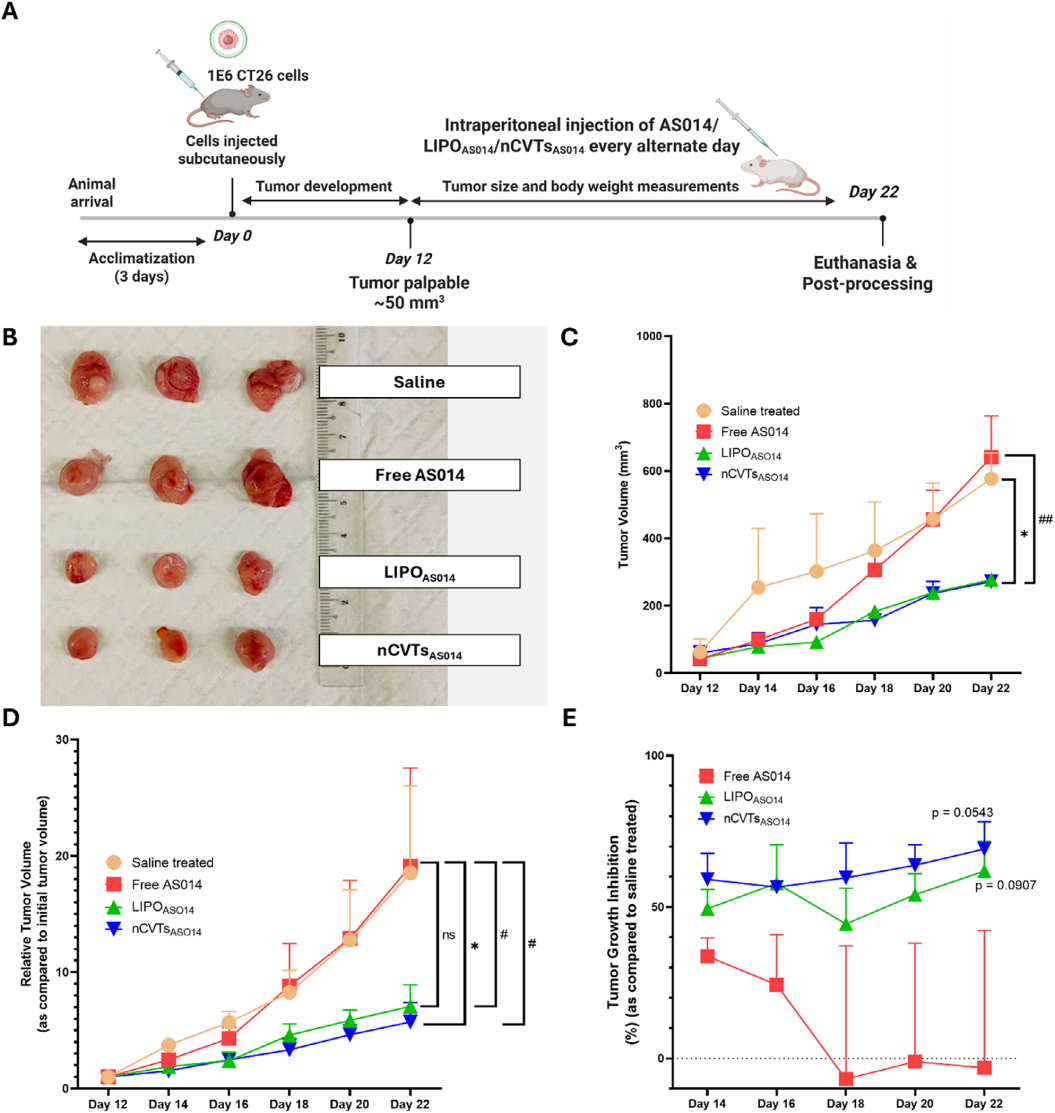
In vivo therapeutic efficacy of nCVTs_AS014_ as compared to LIPO_AS014_ and free AS014. A) Schematic illustrations on the in vivo CT26 tumor mice model. B) Images of the tumor after harvest at Day 22. C) Tumor volume of the mice from different experimental groups throughout treatment. D) Relative tumor volume (as compared to the start of treatment on Day 12) of the mice from different experimental groups throughout treatment. E) Tumor growth inhibition (as compared to saline treatment) of the mice from different experimental groups throughout treatment. *N* = 3, data given as mean ± SEM. *p < 0.05 as compared to saline treatment, *p* < 0.05, ## *p* < 0.01 as compared to free AS014 treatment.

Further evaluation was conducted on the CT26 mice colorectal tumor model (**Figure** **5**A). Upon tumor induction, nCVTs_AS014_, LIPO_AS014,_ and free AS014 (at an ASO concentration of 5 mg k^−1^g) were injected intraperitoneally every alternate day. Expectedly, free AS014 treatment was unable to exert a significant therapeutic effect, justifying the necessity for a drug delivery carrier for NATs like ASO (Figure 5B,C). Both nCVTs_AS014_ and LIPO_AS014_ were able to inhibit the tumor growth progression. When comparing the relative tumor volume, nCVTs_AS014_ exhibited a significant inhibition over the treatment period as compared to the saline treatment (Figure 5D) (*p* = 0.0316). With our cationic nCVTs, AS014 was successfully delivered to the tumor and exerted its intended anti‐cancer effect to inhibit tumor growth progression (Figure 5E).

Excitingly, our study demonstrates that administering AS014 at a dose of 5 mg k^−1^g (every other day) is well tolerated by the mice, as evidenced by the stable body weights throughout the treatment period (Figure S8A, Supporting Information). To ensure the safety of our dosing regimen, we also measured plasma alanine aminotransferase (ALT) and creatinine levels at the end of the treatment—key indicators of liver and kidney health, respectively. Remarkably, both ALT and creatinine concentrations remained comparable to control levels, indicating that our treatment did not induce any significant liver or kidney toxicity (Figure S8B,C, Supporting Information). While there was no notable distinction in the toxicity profiles of nCVTs_AS014_ and LIPO_AS014_, these encouraging results suggest that our dosing strategy effectively suppresses tumor growth without compromising organ health. Supported by promising in vitro toxicity data, nCVTs_AS014_ could even offer a broader therapeutic window compared to LIPO_AS014_. Moving forward, further studies will be instrumental in fine‐tuning the balance between treatment efficacy and safety, paving the way for optimizing the therapeutic window.

## 3 Conclusion

In this work, we developed a novel hybrid DDS termed as nCVTs for targeted delivery of NATs to cancer cells. We successfully complexed AS014 with nCVTs and demonstrated the preferential targeting of nCVTs toward cancer cells with clear targeting of AS014 into the nucleus. Compared to their synthetic counterpart LIPO, nCVTs displayed improved cellular uptake, better penetration into the nucleus, preferential targeting toward cancer cell lines (CT26 colorectal cancer cells) and improved biocompatibility. nCVTs_AS014_ preserved their anticancer efficacy when tested in a colorectal murine model, while showing minimal toxicity at the dose evaluated. Taken together, nCVTs represent a viable new platform for the robust delivery of NATs into target cells.

Additionally, nCVTs demonstrated advantages over other nanoparticle systems, such as liposomes, in terms of the lack of PEGylation (which is associated with complement activation and accelerated blood clearance upon repeated administrations) and immune rejection. Indeed, preliminary in vitro and in vivo studies showed minimal immune activation, with lower nitric oxide and cytokine responses as compared to liposomes.^[^ ^13^, ^14^ ^]^

These findings collectively support the translational potential of nCVTs as a next‐generation delivery system for nucleic acid therapeutics. nCVTs combine the biocompatibility, lack of immunogenicity and targeting capabilities of cell‐derived systems with the tunability of synthetic nanocarriers, offering a promising and translatable platform for targeted drug delivery.

## Conflict of Interest

The authors declare no conflict of interest.

## Supporting information



Supporting Information

## Data Availability

The data that support the findings of this study are available from the corresponding author upon reasonable request.
