# Supplementary material for: Delivery of Antisense Oligonucleotides Using the Nano‐Cell Vesicle Technology System (nCVTs) for Targeted Cancer Therapy
Source: Small. 2025 Dec 16;22(8):e09094. doi: 10.1002/smll.202509094 (PMC12877987; doi:10.1002/smll.202509094)
Supplement: Supplementary file 1 — Supporting Information [file SMLL-22-e09094-s001.docx]

Supporting Information

Delivery of antisense oligonucleotides using the nano-Cell Vesicle Technology system (nCVTs) for targeted cancer therapy

Yi Hsuan Ou#, Wei Heng Chng#, Ram Pravin Kumar Muthuramalingam#, Prathyusha Raghunathan, Nichakan Khunkitchai, Choon Keong Lee, Jae Ha Jun, Zi Xiu Ng, Phua Tian Xin, Wei Jiang Goh, Jiong-Wei Wang, Bertrand Czarny*

Yi Hsuan Ou, Wei Heng Chng, Ram Pravin Kumar Muthuramalingam, Prathyusha Raghunathan, Nichakan Khunkitchai, Choon Keong Lee, Jae Ha Jun, Zi Xiu Ng, Phua Tian Xin, Wei Jiang Goh

Department of Pharmacy and Pharmaceutical Sciences, Faculty of Science, National University of Singapore, 18 Science Drive 4, Singapore.

Jiong-Wei Wang

Department of Surgery, Yong Loo Lin School of Medicine, National University of Singapore, 1E, Kent Ridge Road, NUHS Tower Block, Singapore 119228.

Bertrand Czarny

School of Materials Science and Engineering, College of Engineering, 50 Nanyang Avenue, Block N4.1, Singapore 639798

E-mail: bczarny@ntu.edu.sg

#Equal contribution


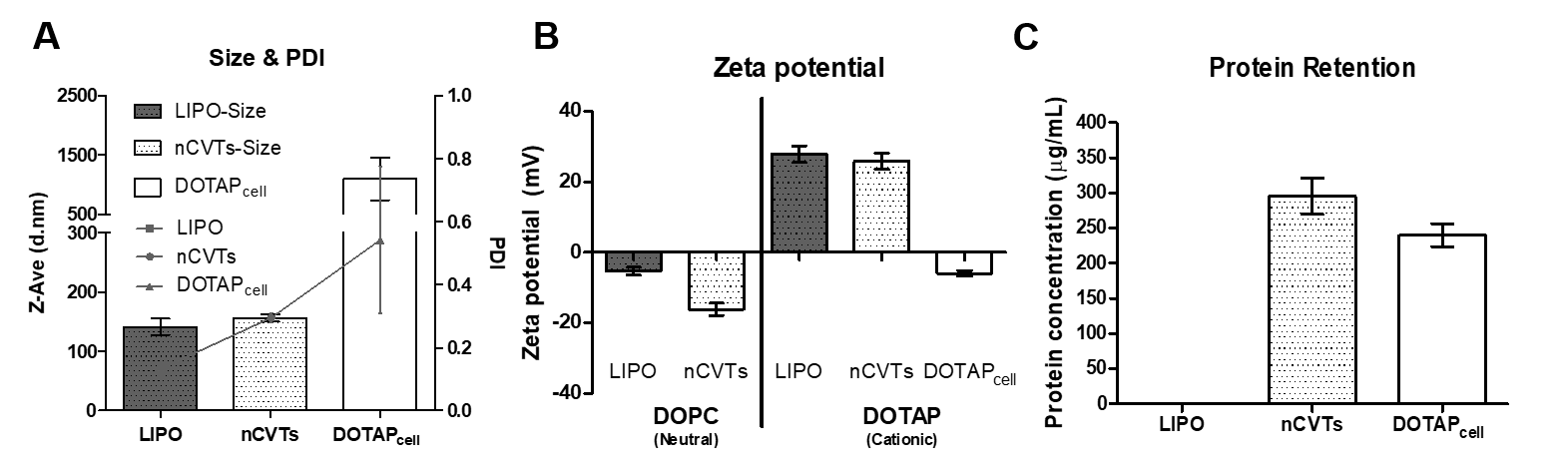


**Figure S1**. Comparison of DOTAP-cell hybrid (DOTAP_cell_) with cationic nCVTs and LIPO. (A) Hydrodynamic size and polydispersity index (PDI) of cationic formulations. Both cationic LIPO and nCVTs have size below 200 nm whereas the DOTAP_cell_ have much larger size. DOTAP_cell_ have much higher PDI than the other two formulations, implying a more polydispersed population. (B) Zeta potential of respective formulations. DOTAP_cell_ have a negative zeta potential despite DOTAP (a cationic lipid) was used in the production. (C) Protein concentrations of respective formulations. The protein concentrations of DOTAP_cell_ have similar protein concentrations as cationic nCVTs.


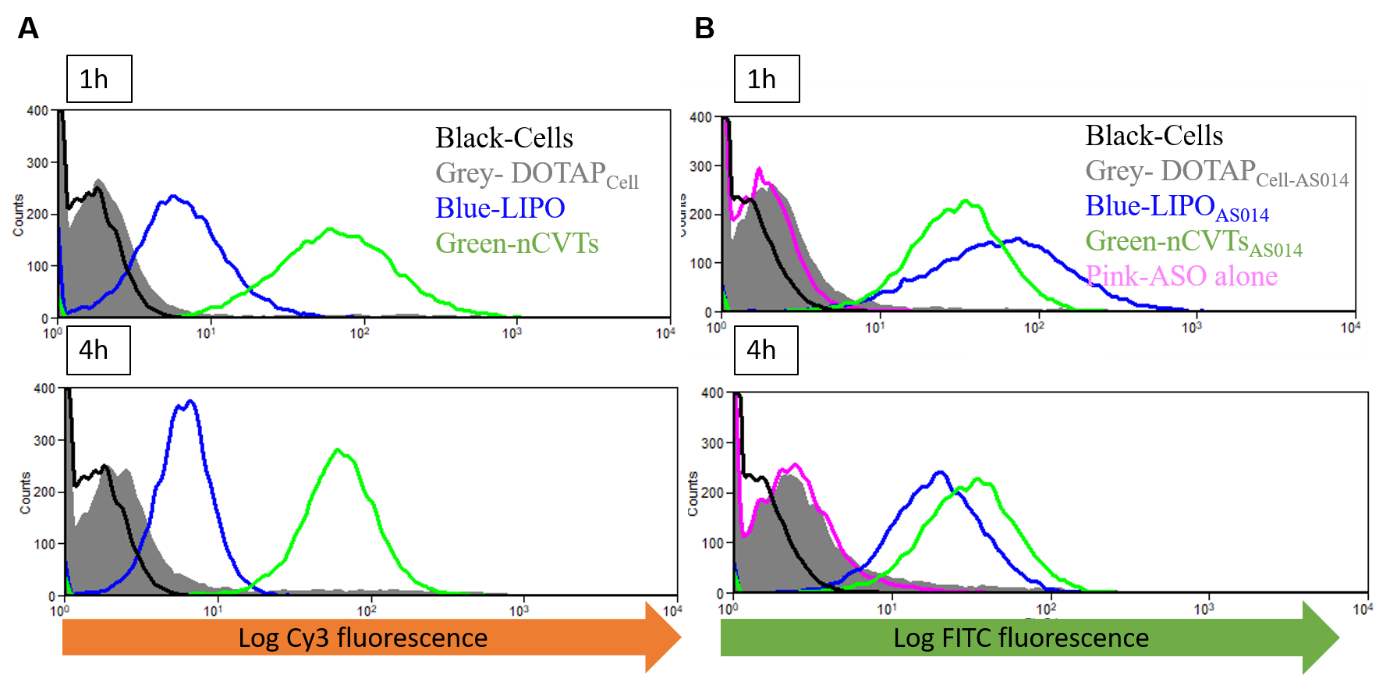


**Figure S2**. Cellular uptake of cationic formulations and ASO. FACS quantitative analysis of (A) cellular uptake of LIPO (blue), nCVTs (green) and DOTAP_cell_ (grey); and (B) uptake of ASO by cells treated with AS014 alone and AS014 loaded formulations at respective 1-hour and 4-hour time points.


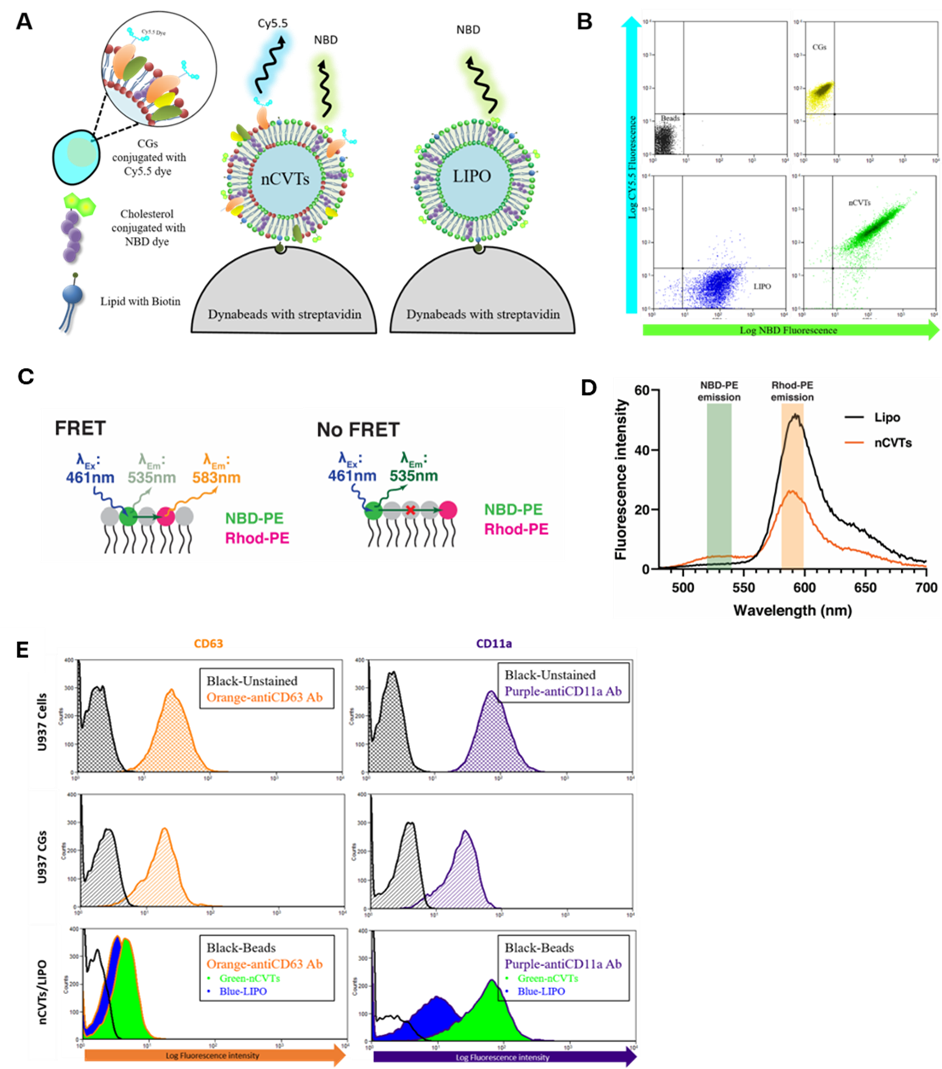


**Figure S3.** Proof of fusion and preservation of protein makers on nCVTs. (A) Schematic illustration of the experimental set up for proof of fusion assay. (B) Flow cytometry analysis of Dynabeads, CGs, and LIPO/nCVTs on Dynabeads. CGs were tagged with Cy5.5; Cy5.5 tagged CGs were subsequently used to produce nCVTs. Thus, these biotinylated nCVTs have both fluorescence signals from CGs (Cy5.5) and lipids (NBD); whereas biotinylated LIPO have only NBD signals. (C) Overview of FRET assay. When the donor NBD fluorophore is excited at λ= 461 nm, NBD emission at λ= 535 nm excites nearby rhodamine acceptor fluorophore which emits at λ= 583 nm. Incorporation and fusion of CGs cell membranes within the lipids increase the distance between the donor and acceptor fluorophore, reducing the FRET effect. (D) Emission spectrum ranging from 480 nm-700 nm of FRET-labelled nCVTs and LIPO. (E) Flow cytometry analysis of protein markers on nCVTs on beads. Parental U937 cells and CGs were used as positive controls. Unstained samples, beads and LIPO were used as negative controls.


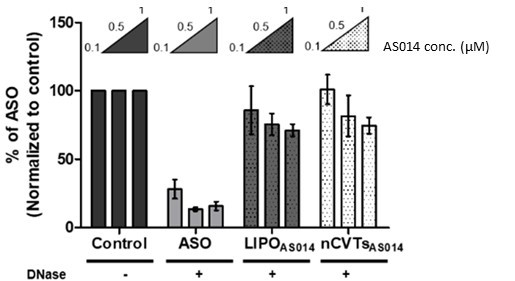


**Figure S4.** Protection assay of free ASO and ASO loaded onto liposomes and nCVTs against DNase I after incubation at 3 concentrations (0.1, 0.5 and 1 µM) for 1 hour. Free ASO was used as a control (n=3).

**Figure S5.** Cell viability of the formulations: nCVTs vs. nCVTs_AS014_ and LIPO vs LIPO_AS014_ normalized based on their respective lipid concentrations.

**
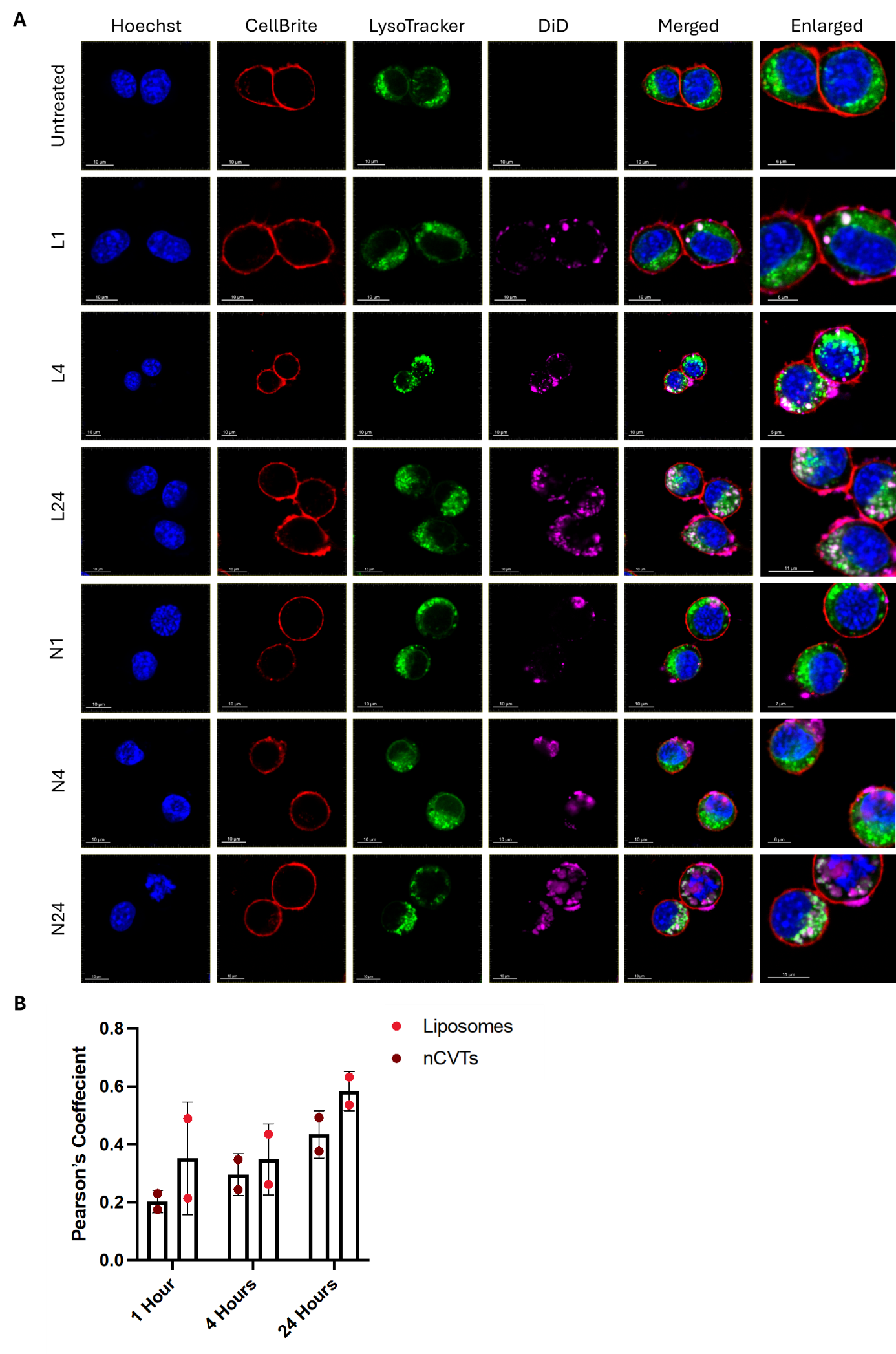
**

**Figure S6.** (A) Confocal images of CT26 cells treated with respective formulations (LIPO or nCVTs) for 1 hour, 4 hours and 24 hours respectively. L1, L4, and L24 denote CT26 cells incubated with LIPO for 1 hour, 4 hours, and 24 hours respectively while N1, N4, and N24 denote CT26 cells incubated with nCVTs for 1 hour, 4 hours, and 24 hours respectively. (B) Pearson correlation coefficient of colocalization of LIPO or nCVTs with lysosomes at 1 hour, 4 hours and 24 hours (N=2, biological replicates).


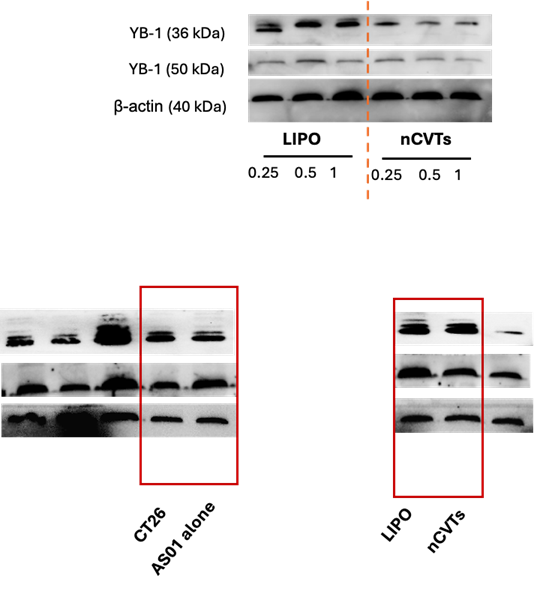


**Figure S7.** Original images of blots.


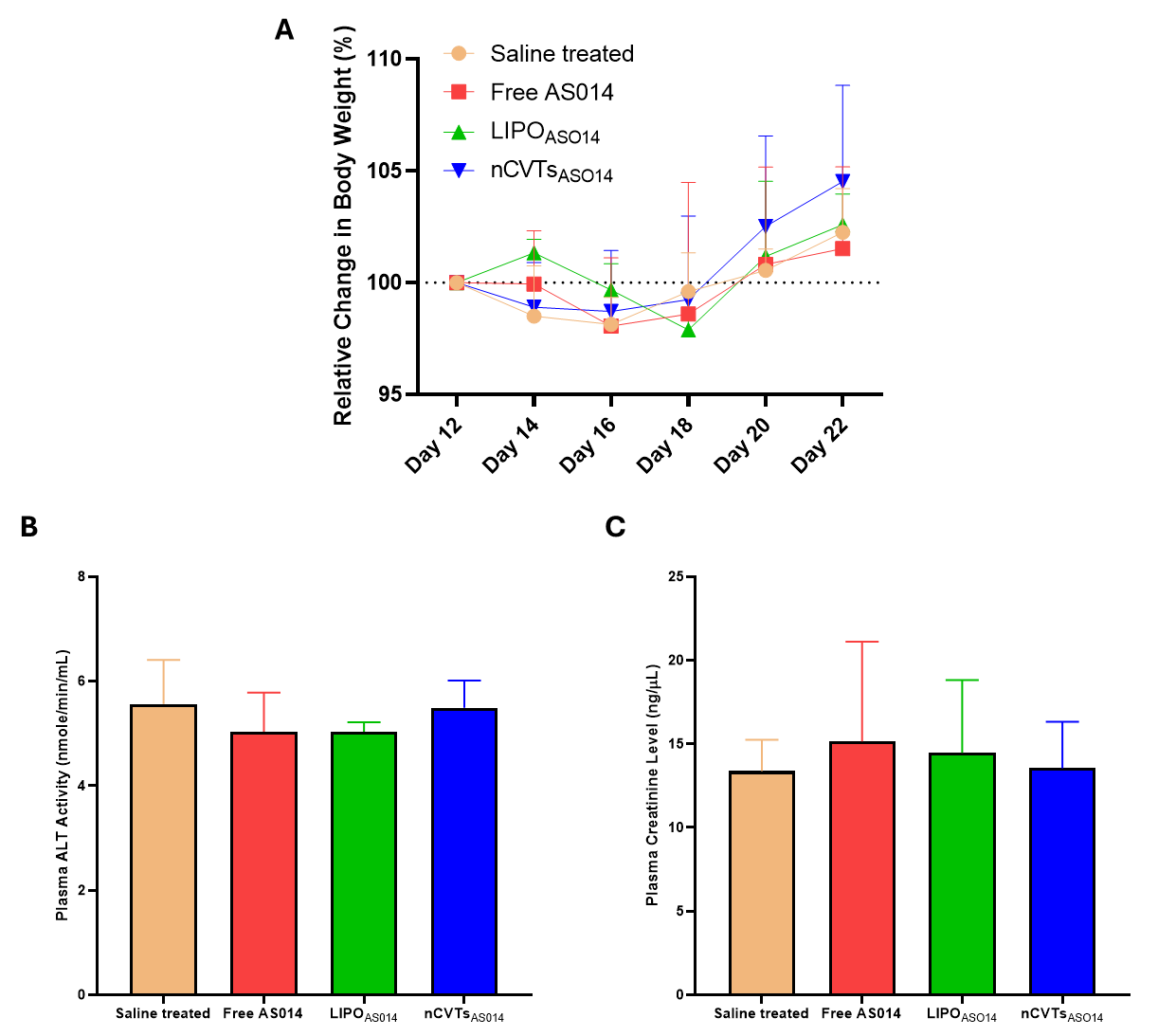


**Figure S8.** Toxicity profile of AS014-loaded formulations (nCVTs_AS014_ and LIPO_AS014_) and free AS014. (A) Body weight of mice during treatment. (B) Plasma ALT concentrations and (C) Creatinine concentrations at the end of treatment.

**Methods**

**Materials**

1,2-dioleoyl-3-trimethylammonium-propane (DOTAP), and cholesterol used in the production of nCVTs and LIPOs were obtained from Avanti Polar Lipids. All antibodies used in this study and protease inhibitor cocktail were purchased from Abcam. Filter membranes used during extrusion were purchased from GE Healthcare Life Sciences. Cyanine 3 N-hydroxysuccinimide) (Cy3-NHS) monoester was obtained from Kerafast and used as per manufacturer’s recommendations. Sodium dodecyl sulfate (SDS), DNase I and ethylendiaminetetraacetic acid (EDTA) were obtained from ThermoFisher Scienctific. The oligonucleotides, AS014 and AS014 tagged with 6-Carboxyfluorescein (FAM) or Cyanine7 (Cy7) were synthesized by Integrated DNA Technologies, Inc. (IDT).

**Cell Culture**

HEK293 and CT26 cells were obtained from American Type Culture Collection (ATCC). U937 cells were a kind gift from Associate Professor Gigi Chiu, National University of Singapore (NUS). CT26 and U937 cells were cultured in RPMI-1640 (Roswell Park Memorial Institute) supplemented with 10% fetal bovine serum (FBS), while HEK293 cells were cultured in DMEM (Dulbecco’s Modified Eagle Media) supplemented with 10% FBS. All cells were maintained in a 5% CO_2_ incubator at 37°C.

**Cell Ghosts (CGs) production**

CG production method was adopted from our earlier work^1^. Briefly, U937 cells were harvested at 70% confluency and resuspended in a hypotonic solution (PBS/sucrose) that consisted of 0.25× phosphate buffer saline (PBS, pH 7.4) and 0.06% w/v sucrose. The cell dispersion was incubated at room temperature for 24 hours, and subsequently resuspended and incubated in 1× PBS/sucrose solution for 24 hours. CGs were then harvested in 1× PBS/sucrose and stored at 4°C for further experiments, the number of CGs were determined by hemocytometer with trypan blue staining. Protease inhibitor cocktail was added throughout the experiment according to manufacturer’s protocol.

**Cationic nCVTs and LIPO Production**

Lipid thin film was prepared by dissolving 2 mg of DOTAP lipid and Cholesterol (in 7:3 mol%) in 1mL of chloroform and evaporating the solvent via rotary evaporation under pressure. 1 × 10^7^ of prepared CGs were used to rehydrate the lipid film. The resulting solution was vortexed and sonicated for up to 1 hour on ice. The dispersion was then extruded through a 0.4 µm filter membrane to obtain cationic nCVTs (**Figure S9**). Cationic LIPOs were produced in a similar fashion, but without the addition of CGs. To produce Cy 3-labelled vesicles, Cy3 NHS monoester was added during the lipid thin film formation, which was subsequently used for production of nCVTs/LIPOs, the unbound free dye was then removed by dialysis overnight.

**Figure S9**. Schematic overview of the key steps in the production of cationic nCVTs^2^. Figure prepared using Biorender.com.

**Size, Zeta Potential and Protein Quantification**

Size and zeta potential of the produced cationic nCVTs and LIPO were measured using Malvern Zetasizer. Protein concentration was quantified by standard BCA assay kit with the addition of 2% SDS to correct for lipid interference^3^.

**Proof of fusion assay and protein marker analysis**

For flow cytometry analysis of nCVTs and liposomes, biotinylated nCVTs and liposomes were incubated with streptavidin-beads overnight at 4°C to allow the binding between streptavidin (on the beads) and biotin (on nCVTs/liposomes). The beads were then centrifuged at 3000 G for 10 minutes and wash thrice with cold PBS to remove unbounded nCVTs/liposomes. Primary antibodies (1:500 dilution) were added to the samples and incubated for 1 hour at 4°C before washing again with cold PBS. The beads were then incubated with secondary antibody (1:1000) for 1 hour at 4°C. After the incubation, the beads were washed thrice with cold PBS to remove the excessive/unbound antibody and analyzed using a flow cytometry analyzer (BD LSRForetessa Flow Cytometry analyzer, US).

**Fluorescent Resonance Energy Transfer (FRET) Assay**

Fluorescent resonance energy transfer (FRET) labelled nCVTs and liposomes were prepared similarly as described before but with the addition of 0.3 mol% 1,2-dipalmitoyl-sn-glycero-3- phosphoethanolamine-N-(7-nitro-2-1,3-benzoxadiazol-4-yl) (NBD) and 0.7 mol% 1,2- dioleoyl-sn-glycero-3-phosphoethanolamine-N-(lissamine rhodamine B sulfonyl). The fluorescence donor, NBD, was excited at 461 nm and emission spectrum between 480 nm and 700 nm was measured using Varioskan LUX microplate reader.

**Preparation of ASO-loaded complexes**

ASO was added into the respective cationic formulations (LIPO and nCVTs) and incubated at room temperature for 30 minutes for complexation. The complexation and stability of ASO to cationic nCVTs/LIPO were confirmed by electrophoretic retardation experiments. Various ratios of ASO to nCVTs/LIPO (total 10 μL of complexes) were mixed with loading buffer (5×) before being loaded onto 2% agarose gel (w/v) in TAE buffer (40 mM Tris/HCl, 1% acetic acid, 1 mM EDTA, pH 7.4). The gel containing SABR green dye was used to allow visualization of nucleic acid. The electrophoresis was carried out at a constant voltage of 80 V for 45 min. The gel was then imaged using a gel documentation system (ChemiDoc MP, Bio-Rad).

**DNase I protection assays**

Various ratios of ASO to nCVTs/LIPO were prepared as described. DNase I (1 unit per μg of DNA) was added to the respective samples before incubating at 37°C for 30 minutes and 1 hour, respectively. EDTA (500mM) was added to stop the action of DNase I after the incubation. SDS (to a final concentration of 1%) was then used to release DNA from the complexes before analysis using gel electrophoresis as described above. The integrity of the ASO in each formulation was compared with untreated ASO as a control.

**MTT (3-(4,5-dimethylthiazol-2-yl)-2,5-diphenyltetrazolium bromide) Cell Viability Assay**

1 × 10^4^/well HEK293 or CT26 cells were seeded in 96 well plates and incubated overnight prior to the treatment with different concentrations of empty cationic nCVTs and LIPO (normalized according to the cationic lipid content), or AS014 loaded vesicles (nCVTs_AS014_ and LIPO_AS014_, normalized to the concentration of AS014). AS014 alone was also used as control. After incubating for 72 hours at 37°C, the culture medium was then replaced with 100 µL of MTT (0.5 mg/mL) and incubated for 1 hour at 37°C. Dimethyl sulfoxide (DMSO) was used to dissolve the formazan crystals, and the absorbance was measured using microplate reader at 570 nm.

**Cellular Uptake Assay of Cationic nCVTs and LIPO**

Fluorophore labelled nCVTs and LIPO were produced as described earlier for cellular uptake study. Briefly, 2 × 10^5^/well CT26 cells were seeded in 6 well plates and incubated overnight at 37°C. The cells were then treated with respective formulations: empty nCVTs and LIPO (normalized according to the cationic lipid content), or AS014 loaded vesicles (nCVTs_AS014_ and LIPO_AS014_) and AS014 alone (normalized to the concentration of AS014) for 6 hours. Cells were then harvested and washed with PBS thrice before analysis by BD LSR Foretessa Flow Cytometry analyzer. Cells were also grown on confocal dishes overnight before being treated with respective formulations: nCVTs_AS014,_ LIPO_AS014_ and AS014 alone for 6 hours. The cell culture media were removed, and the cells were fixed with 4% paraformaldehyde for 15 minutes at room temperature and washed with PBS once before staining with Hoechst 33342 and CellMask DeepRed according to manufacture protocol. Imaging was conducted using Nikon Eclipse Ti inverted fluorescence microscope and captured with DS-Qi2 camera with similar exposure time. NIS-Elements Basic Research software was used to quantify fluorescence intensity.

**Lysosome Colocalization Assay**

To produce DiD-labelled vesicles, 1,1′-dioctadecyl-3,3,3′,3′- tetramethylindodicarbocyanine, 4-chlorobenzenesulfonate salt (DiD) was added during the lipid thin film formation, which was subsequently used for production of nCVTs/LIPOs. 1 × 10^4^/well CT26 cells were seeded in 8-well confocal slides and incubated overnight at 37°C. The cells were then treated with DiD-labelled nCVTs or LIPO for 1, 4 and 24 hours respectively. The cell culture media were removed, and the cells were washed with PBS thrice before staining with the following dyes at 1X working concentration: Hoechst 33342 (ThermoFisher Scientific, USA), CellBrite (Biotum, USA), and LysoTracker^TM^ Green (ThermoFisher Scientific, USA). The cells were imaged using FLUOVIEW FV3000 confocal microscope (Olympus) and the images were analyzed using Imaris v9.7 (Oxford Instruments).

**Western Blot**

The expression of YB-1 protein after the treatment with AS014, was evaluated using Western blot. 8 × 10^5^/well CT26 cells were seeded in 6 well plates and incubated overnight at 37°C. The cells were treated with respective formulations: nCVTs_AS014_/LIPO_AS014_/AS014 alone for 6 hours at 37°C. The media were then replaced with fresh medium for further incubation of 72 hours. Proteins were extracted from the cells with RIPA buffer (1% v/v NP-40, 0.5% w/v sodium deoxycholate and 0.1% w/v SDS in 1× PBS) supplemented with protease and phosphatase inhibitors (50 mM sodium fluoride, 2 mM sodium orthovanadate, 1 mM phenylmethane sulfonyl fluoride (PMSF) and 4 µg/mL aprotinin), before being quantified using Bradford protein assay reagent kit (Bio-Rad Laboratories). Protein samples were separated using 10% Sodium dodecylsulfate-polyacrylamide gel electrophoresis (SDS-PAGE) under reducing conditions and then transferred to Millipore PVDF membranes. The membrane was blocked using 5% BSA, followed by incubation with anti-YB1 antibody at 4oC overnight. Horseradish peroxidase (HRP)-conjugated secondary antibody was used to detect the primary antibody, and the blot was developed with Clarity Western ECL blotting reagent (Bio-Rad). β-actin was stained as a housekeeping protein.

***In vivo* study using mouse syngeneic model**

All animal experiments were approved by the Institutional Animal Care and Use Committee (IACUC) at the National University of Singapore (NUS: protocol number R19-0769). Female BALB/c mice (5-6 weeks) were engrafted subcutaneously with 1 × 10^6^ CT26 mouse colon cancer cells at the flank. The tumor was allowed to develop for 12 days before injecting the sample intraperitonially on every alternate day.

Once the CT26 tumor is palpable, the mice were injected with respective treatments (LIPO_AS014_, nCVTs_AS014_, free AS014 or saline) intraperitoneally. The tumor size and body weight of the animals were measured every alternate day. At the end of the experiment, the mice were euthanized, and the tumors were harvested for further analysis. Blood was collected by cardiac puncture for further analysis. Serum ATL and creatinine were measured using the Colorimetric Assay kits obtained from Sigma Aldrich (US).

**Statistical Analysis**

All statistical analysis was done via GraphPad Prism 5 (GraphPad Software). One-way ANOVA with Bonferroni’s post hoc test was performed. All data were presented as mean ± SD. P < 0.05 was significant.

**References**

1. Goh, W. J.; Zou, S.; Czarny, B.; Pastorin, G., nCVTs: a hybrid smart tumour targeting platform. *Nanoscale* **2018,** *10* (15), 6812-6819.
2. Zhang, H., Thin-Film Hydration Followed by Extrusion Method for Liposome Preparation. In *Liposomes: Methods and Protocols*, D'Souza, G. G. M., Ed. Springer New York: New York, NY, 2017; pp 17-22.
3. Brown, R. E.; Jarvis, K. L.; Hyland, K. J., Protein measurement using bicinchoninic acid: elimination of interfering substances. *Analytical Biochemistry* **1989,** *180* (1), 136-139.
